# Sufficient competence in community elderly care? Results from a competence measurement of nursing staff

**DOI:** 10.1186/s12912-016-0124-z

**Published:** 2016-01-14

**Authors:** Pia Cecilie Bing-Jonsson, Dag Hofoss, Marit Kirkevold, Ida Torunn Bjørk, Christina Foss

**Affiliations:** Department of Nursing Science, Institute of Health and Society, Faculty of Medicine, University of Oslo, Postbox 1130 Blindern, 0318 Oslo, Norway

## Abstract

**Background:**

Multi-morbidity, poly-pharmacy and cognitive impairment leave many old patients in a frail condition with a high risk of adverse outcomes if proper health care is not provided. Knowledge about available competence is necessary to evaluate whether we are able to offer equitable and balanced health care to older persons with acute and/or complex health care needs. This study investigates the sufficiency of nursing staff competence in Norwegian community elderly care.

**Methods:**

We conducted a cross-sectional survey of 1016 nursing staff in nursing homes and home care services with the instrument “Nursing Older People – Competence Evaluation Tool”. Statistical analyses were ANOVA and multiple regression.

**Results:**

We found that nursing staff have competence in all areas measured, but that the level of competence was insufficient in the areas nursing measures, advanced procedures, and nursing documentation. Nursing staff in nursing homes scored higher than staff in home care services, and older nursing staff scored lower than younger nursing staff.

**Conclusions:**

A reason for the relatively low influence of education and training on competence could be the diffuse roles that nursing staff have in community elderly care, implying that they have poor standards against which to judge their own competence. Clearer role descriptions for all groups of nursing staff are recommended as well as general competence development in geriatric nursing care.

## Background

This article presents results from a survey measuring the competence of community-based nursing staff working with older patients. The survey was the first full trial of a new competence measurement instrument labelled “Nursing Older People – Competence Evaluation Tool” (NOP-CET). The questionnaire was developed for three groups of nursing staff; registered nurses (RN), assistant nurses (AN) and assistants, working in nursing homes and home care services (defined as community elderly care).

### The patients

In Europe the patient populations in receipt of community elderly care are characterised by multi-morbidity, poly-pharmacy and/or cognitive impairment [1–5]. Multi-morbidity refers to the coexistence of two or more conditions in a patient [6], is found to negatively influence quality of life and the ability of self-care [7], and is associated with significant increases in adverse events, hospitalisations and cost of care [8]. Poly-pharmacy is defined as the consumption of multiple medications or administration of more medications than clinically indicated [9], and is increasing among elderly people [10–12]. Several studies indicate that inappropriate drug use is a major reason for impaired health and function in the elderly [13–15]. Additionally, a large proportion of patients in receipt of community elderly care suffer from cognitive impairment in terms of declining memory and other cognitive abilities. Almost 83 % of all patients admitted to nursing homes in Norway suffer from dementia [16], of which 66 % have clinically significant neuropsychiatric symptoms [17, 18]. In sum, multi-morbidity, poly-pharmacy and cognitive impairment leave many old patients in a frail condition with a high risk of adverse outcomes if proper health care is not provided and performed [19]. A well-educated staff that can competently meet the needs of these patients is therefore essential [20, 21].

### The staff

Studies indicate that better quality of care, improved patient outcomes, and fewer adverse events are associated with higher levels of registered nurse staffing in health care [22–27]. Still, we know that approximately 30 % of the nursing staff in Norwegian community elderly care are assistants without any formal health care training [28], approximately 60 % of the staff – the assistant nurses – are qualified through a degree from upper secondary school [29], and that most of the staff, including the RNs, have not had the opportunity to develop their competence in accordance with increasing job demands [30]. Despite different lengths in education and training, the roles of RNs, ANs, and assistants in Norwegian community elderly care are fairly similar [31]; there is little distinction between the roles and responsibilities of different types of nursing staff [32, 33].

Despite efforts to enhance the quality of community elderly care, there are many reports of inadequate health care in terms of unmet needs, adverse events, and other threats to quality of care [1, 34–38]. Such reports indicate the need to investigate whether the competence available in community elderly care is sufficient to meet complex patient needs. A literature review of the role RNs play in home care revealed that there were no studies investigating the competence of nursing staff in Norwegian community elderly care. Knowledge about available competence is necessary to evaluate whether we are able to offer equitable and balanced health care to older persons with acute and/or complex health care needs [20].

Our research questions were:

- What is the competence of nursing staff in community elderly care?
- What influences competence as measured?

## Conceptualisation of competence

**Fig. 1:**
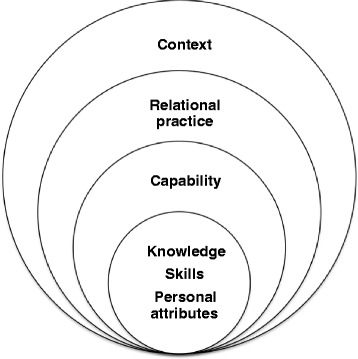
Conceptualisation of competence

Competence is a concept that has been used to cover several meanings in the nursing literature [39], but usually in reference to the execution of tasks and duties expected of professionals [40]. Our conceptualisation of competence is inspired by Eraut [40] as well as more socio-cultural conceptualisations as represented by Edwards [41]. Eraut discussed the importance of including capability into a conceptualisation of competence, as capability provides a basis for future competence (i.e. a knowledge-base), and thus expands former conceptualisations which have focused on performance as the main attribute of competence [40]. Edwards focuses on the relational aspects of competence, meaning that the individual competence of a practitioner is inherently bound to the competence of other practitioners [41]. Competence is a collective activity, and the goal within a workplace should be that nursing staff are able to reciprocally strengthen each other’s competence so that the amount of collective competence is larger than the sum of individual competence. Competence as measured in the NOP-CET therefore includes a mix of approaches in order to grasp individual competence in terms of knowledge, skills, and personal abilities, as well as relational and contextual aspects of competence in community elderly care. Our conceptualisation can be illustrated as in Fig. 1.

## Method

### Instrument description and development

The NOP-CET is a comprehensive questionnaire measuring competence in community elderly care. The questionnaire contains a total of 65 items. There are two main types of items: items with Likert-type scales (all except one item use a four-point scale) and items with dichotomous scores (correct/wrong). Some items ask for self-evaluation, others are in multiple-choice format. The NOP-CET was administered electronically by the online tool “Questback”.

The development of the NOP-CET had three phases, of which the first was a literature review of competence measurement instruments, which revealed that no existing instruments measured the competence required of nursing staff in current community elderly care [42]. In phase two we consulted 42 experts on community elderly care, in three rounds, in order to reach a consensus on the most relevant items for measuring the competence expected of nursing staff in community elderly care [43]. We found that the most relevant competence could be covered within ten categories: health promotion and disease prevention, treatment, palliative care, ethics and regulation, assessment and taking action, covering basic needs, communication and documentation, responsibility and activeness, cooperation, and attitudes toward older people. Questionnaire items were developed from these ten categories. The questionnaire was pilot-tested on 26 RNs for acceptability, comprehensiveness, relevancy, and unambiguity. The third and final phase consisted in a trial of the NOP-CET on a sample of 1016 RNs, ANs, and assistants, on which the results are reported here.

The NOP-CET was evaluated to be appropriate as it showed good content and construct validity, reliability, precision, interpretability, acceptability, and feasibility [44]. Principal components analysis with direct oblimin rotation and retention of factors with eigenvalues > 1 produced 28 clinically meaningful factors that were used to form sum-variables for analysis of competence. The sum-variables with corresponding questionnaire items and factor loadings are displayed in Tables 1, 2 and 3.

**Table 1:** Table of sum variables measuring knowledge

Knowledge sum variables with associated items and their factor loadings					
Palliative care		7.4 Have the wound assessed by physician	-.50	Diabetes treatment	
20.1 Assess a patient’s pain	.80	7.8 How to document wound care	-.47	12. Patient case concerning hypoglycaemia	-.57
20.2 Assess effectiveness of pain relieving medication	.74	7.1 Give pain relief before wound care	-.40	11. What type of insulin is Insulatard	-.45
20.10 End of life care	.65	Nursing measures		10. What is the desired blood sugar level of diabetes patients	-.39
20.11 How to communicate about death with patient and family	.63	16.7 Patient has reduced appetite and food intake	.69	Medication calculation	
20.5 Assess measures against dyspnoea, nausea, and obstipation	.60	16.6 Patient’s skin has rash, wounds, is red or itchy	.66	14. How many tablets should the patient have in total	.78
20.9 Assure a patient’s own wishes surrounding death	.59	16.9 Patient has pain and discomfort in mouth	.66	13. How many ml is the dosage	.76
20.4 Use non-medical pain relief methods	.45	17.4 Patient is more tired during the day	.61	Involving physician	
20.3 Assess need for alternative medical pain relief methods	.36	17.7 Patient has lost interest in keeping home in order, sleeps in chair instead of bed	.57	16.11 Patient has much fresh blood in stool	.67
Acute help		16.2 Patient coughs, has increased saliva, and respiration frequency above 20/min	.49	16.5 Patient is substantially dehydrated	.53
17.3 Patient has symptoms of partial paralysis	.69	16.3 Patient has irregular pulse increased more than 20/min in last two days	.38	16.10 Patient is incontinent for urine, stings when urinates	.42
17.6 Patient has newly occurred chest pain	.62	Deficiencies in sight and hearing		16.8 Patient not able to eat	.41
17.5 Patient has changes in sight, hearing, speech, and comprehension	.54	5.4 How to book time for sight- and hearing control	.55	17.1 Patient has increased needs to full care over last two days	.30
17.2 Patient has fallen two times previous week	.42	5.2 How to communicate with patients with hearing deficiencies	.54	Health promotion	
17.8 Patient has short attention span and delusions	.34	5.3 How to facilitate light for patients with sight deficiencies	.54	2. How to find a patient’s resources and preferences	-.68
Wound care		5.1 How to change batteries and clean hearing aids	.51	1. How to find meaningful activities for a patient	-.64
7.2 How to perform hand hygiene before wound care	-.98	Fall prevention		3. What rights a patient has to participation/ empowerment	-.42
7.7 How to assess the skin around the wound	-.98	6.4 Patient goes to toilet at least once an hour	.58	Newer palliative measures	
7.3 How to perform hand hygiene after wound care	-.97	6.2 Patient seems agitated and restless	.55	20.6 Use the tool ESAS^a^	.55
7.6 How to assess changes in a wound	-.96	6.3 Patient’s sight is too poor to perform all activities by himself	.53	20.8 Use the tool LCP^b^	.49
7.5 How to follow the wound care procedure	-.96			20.7 Transfer a palliative patient to other treatment level	.39

^a^Edmonton Symptom Assessment System, ^b^Liverpool Care Pathway

**Table 2:** Table of sum variables measuring skills

Skills sum variables with associated items and their factor loadings					
Patient observations		21.12 Subcutaneous injection as e.g. fragmin	-.33	23.1 Exploit patient bed’s mechanical function	.66
21.24 Take pulse	.88	Nursing documentation		23.4 Use appropriate tools for body mechanics	.56
21.23 Take blood pressure	.80	44.4 Update nursing plan	-.83	23.2 Use sliding mat for moving patient in bed	.45
21.27 Take temperature	.50	44.3 Develop nursing plan	-.83	Simple procedures	
21.26 Count respiration frequency	.50	44.6 Register patient in national register	-.62	21.25 Weigh a patient	.48
Advanced procedures		44.5 Write nursing report for dismissal/referral	-.60	21.11 Perform ostomy care	.41
21.16 Use of central venous catheter	-.79	Treatment		Make oneself understood	
21.15 Handle intravenous pumps	-.77	21.2 Inject insulin	-.72	48.2 Make yourself understood around colleagues	.72
21.18 Administer intravenous medication	-.76	21.1 Monitor blood glucose	-.72	48.1 Make yourself understood around patients	.55
21.17 Puncture via Venous Access Port	-.71	21.7 Dispense medication	-.50	Electronic communication	
21.5 Insert permanent urinary catheters	-.59	21.6 Apply/change transdermal analgesic patch	-.39	44.7 Send electronic message to physician	-.72
21.4 Insert intermittent urinary catheter on man	-.55	21.8 Wound care	-.37	44.8 Send electronic message to hospital	-.48
21.13 Intramuscular injection	-.55	21.10 Administer nebulizer treatment	-.36	Patient assessment	
21.19 Handle a drain	-.46			21.28 Assess patient’s urine	.86
21.14 Blood sampling	-.45	Body mechanics		21.29 Assess patient’s stool	.82
21.20 Handle ECG	-.42	23.3 Adjust patient bed to own elbows’ height	.74	21.22 Assess skin of patient	.67
21.3 Insert intermittent urinary catheter on woman	-.38	23.5 Use patient lift	.71	21.21 Assess whether a patient has edema	.55

**Table 3:** Table of sum variables measuring personal attributes

Personal attributes sum variables with associated items and their factor loadings					
Cooperation with physician and next-of-kin		38.4 Need to communicate with physician, but cannot reach	.40	39.5 Get necessary and sufficient information from leader	-.71
39.4 Get necessary and sufficient information from physician	.78	Cooperation with ANs and assistants		37.5 Communicate with leader about patient	-.63
41.4 Physician knows content of your work	.74	40.3 Solve patient problems in cooperation with assistants	.73	41.5 Leader knows content of your work	-.63
37.4 Communicate with physician about patients	.71	41.3 Assistants know content of your work	.61	Cooperation with RN	
40.4 Solve patient problems in cooperation with physician	.68	39.3 Get necessary and sufficient information from assistants	.60	40.1 Solve patient problems in cooperation with RN	-.75
39.6 Get necessary and sufficient information from next-of-kin	.44	39.2 Get necessary and sufficient information from ANs	.56	41.1 RNs know content of your work	-.63
40.6 Solve patient problems in cooperation with next-of-kin	.41	40.2 Solve patient problems in cooperation with ANs	.47	39.1 Get necessary and sufficient information from RN	-.50
37.6 Communicate with next-of –kin about patient	.33	41.2 ANs know content of your work	.37	Communication	
41.6 Next-of-kin knows content of your work	.31	Cooperation concerning documentation		37.1 Communicate with RN about patient	.63
Not being able to reach co-workers		42.2 Sufficient documentation from physician to comprehend a patient’s situation	.78	37.3 Communicate with assistants about patient	.60
38.2 Need to communicate with AN, but cannot reach	.92	42.3 Sufficient documentation from hospital to comprehend a patient’s situation	.75	37.2 Communicate with ANs about patient	.52
38.3 Need to communicate with assistant, but cannot reach	.85	42.4 Sufficient documentation from other services to older people to comprehend a patient’s situation	.65	Attitudes towards elderly	
38.1 Need to communicate with RN, but cannot reach	.84	42.1 Sufficient documentation from own workplace to comprehend a patient’s situation	.50	43.4 Patient case: showing respect when entering someone’s home	.52
38.5 Need to communicate with leader, but cannot reach	.69	Cooperation with leader		43.2 Patient case: showing respect/moral behaviour	.47
38.6 Need to communicate with next-of-kin, but cannot reach	.61	40.5 Solve patient problems in cooperation with leader	-.72	43.3 Patient case: showing humility	.36

### Recruitment and sample

Nine municipalities were invited to take part in the trial of the NOP-CET, and all nine accepted. Different “types” of municipalities were sought to represent different composition of staff, as well as varying degrees of commitment to competence development. The municipalities were also selected to represent different socio-demographic areas; three were urban areas, five suburban, and two rural. Six municipalities agreed to invite all their nursing staff employed in nursing homes and home care services. One municipality agreed to include four home care units, another municipality included two nursing homes. One municipality invited a random sample from all nursing homes and home care services in their municipality. Eight of the participating municipalities are located in the south-eastern part of Norway, while one municipality is in northern Norway. Table 4 shows the sample properties of this study.

**Table 4:** Characteristics of participants, *N* = 1016

Variable		Count	Percent	Variable		Count	Percent
Gender	Female	947	93.2	Workplace	Home care	321	31.6
	Male	44	4.3		Nursing home	553	54.4
	Missing	25	2.5		Other services	108	10.6
Age	18–25	57	5.6		Missing	34	3.3
	26–30	79	7.8	Position size	up to 25 %	46	4.5
	31–40	217	21.4		26–50 %	136	13.4
	41–50	289	28.4		51–75 %	249	24.5
	51–60	261	25.7		76–90 %	193	19.0
	61–70	69	6.8		91–100 %	322	31.7
	Missing	44	4.3		Missing	70	6.9
Group of nursing staff	RN	354	34.8	Position type	Permanent	904	89.0
	AN	528	52.0		Temporary	72	7.1
	Assistant	90	8.9		Missing	40	3.9
	Others^a^	44	4.3				
	N	Mean	SD	Min	Max		
Years at current workplace	983	8.16	7.48	.0	55.0		
Years of experience in community elderly care	992	15.60	10.26	.0	45.0		

^a^Others include physiotherapists, occupational therapists, secretaries, and leaders

The response time was estimated up to one hour. Respondents were nursing staff who were offered one hour off from normal work tasks to fill in the NOP-CET at a computer during working hours. Managers working in the nursing homes and home care services informed their staff of their municipality’s commitment to the study, facilitated response/participation by giving respondents one hour off normal working tasks, provided access to complete the questionnaire at their work place, and generally encouraged all groups of staff to participate. The managers provided us with the e-mail addresses of all nursing staff, who then received an e-mail from us with an invitation and link to the NOP-CET. One municipality could not provide e-mail addresses, therefore letters with the link to the questionnaire were mailed to all nursing staff. Participation in the survey was voluntary and confidential. The participants were informed that filling out the questionnaire was synonymous with informed consent. Research approval from Norwegian Social Science Data Services was obtained on June 3, 2013.

Question no. 50 in the NOP-CET: “In general, to which degree are you competent to give safe health care to older people?” (5-point Likert scale) was considered to be the item that encompassed all aspects of competence necessary in community elderly care. Sample size calculations were performed in order to find the minimum sample size needed to be able to document a difference of 10 % between RNs and ANs, a 50 % difference between RNs and assistants, and a 40 % difference between ANs and assistants on question no. 50, with a maximum risk of committing a Type I error of 5 %, and a Bonferroni correction for multiple comparisons (three groups) [45]. From this follows that we would need 387 RNS, 387 ANs, but no more than 28 assistants. The actual number of nursing staff that responded to the survey were 354 RNs, 528 ANs, and 90 assistants.

Data collection took place between September and December 2013. The questionnaire was initially sent to 3175 nursing staff of which 1016 responded. The response rate varied between the municipalities: the lowest and highest response rates were 15 and 62 %, with a total response rate of 36 %. Once the questionnaire was published on the internet the nursing staff had two weeks to complete the NOP-CET. Two reminders were sent during the last week the questionnaire was open. The NOP-CET was kept open for an additional week for those municipalities that requested this. At completion of the survey all responses were exported electronically to SPSS. No questionnaires were rejected, as the number of missing data was considered acceptable (maximum missing data on an item was 18.3 %).

### Data analysis

**Fig. 2:**
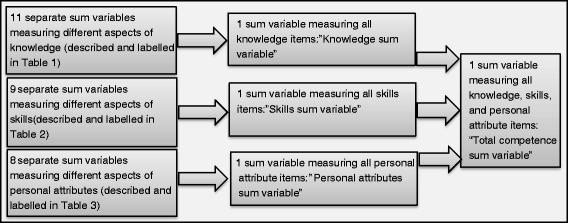
Illustration of the formation of sum variables

Data analysis was performed in SPSS Statistics Version 20. Sum-variables were formed out of the 28 factors produced in the factor analysis that evaluated the construct validity of the NOP-CET [44]: 11 sum-variables measured knowledge (described in Table 1), nine sum-variables measured skills (described in Table 2), and eight sum-variables measured personal attributes including relational and contextual aspects (described in Table 3). The variables that measured knowledge were formed into “Knowledge sum-variable”, the variables that measured skills were formed into “Skills sum-variable”, and the same operation was done for personal attributes and a “Total competence sum variable”. The procedure of forming sum-variables is illustrated in Fig. 2. The sum-variables were treated as continuous variables where equal intervals on the variables represent equal differences in competence. The value 0 (zero) was imputed when a value was missing in the outcome variables (i.e the variables for knowledge, skills, personal attributes, and total competence) in order to avoid casewise deletion, as the survey can be seen as a test, and no value equals no addition to the respondent’s competence score. Count, percentage, mean, and standard deviation (SD) were produced to describe the sample. The level of significance was accepted at *p* <0.05. Confidence intervals (CI) and standard errors (SE) are based on 1000 bootstrap samples as normally distributed errors and homoscedasticity could not be assumed for all variables.

To answer the first research question, “What is the competence of nursing staff in community elderly care?”, we analysed the sum-variables by ANOVA to describe the competence of the three groups of nursing staff.

Research question number two “What influences competence as measured?” was analysed with multiple regression. The outcome variable was “Total competence sum-variable”. The predictor variables were *age* (continuous variable), *gender* (dichotomous variable; Female = 0, Male = 1), *professional group* (four categories: RN/AN/Assistant/Others, three dummies were created), *work place* (three categories: Home care/Nursing home/Other services for elderly, two dummies were created), *type of position* (dichotomous variable; Permanent = 0, Temporary = 1), *job size* (i.e. fraction of full-time employment, e.g. 80 %) (continuous variable), *time at workplace* (i.e. number of years at current work place) (continuous variable), and *time in community elderly care* (i.e. years the person has worked in community elderly care) (continuous variable). Confidence intervals were bootstrapped as normally distributed errors were not assumed, and weighted least squares regression was chosen to overcome eventual problems of heteroscedasticity [45].

## Results

The respondents to the survey comprised 35 % RNs, 52 % ANs, but only 9 % assistants. Most of the respondents were female (93 %) and the majority were employed in permanent positions (89 %) in nursing homes (54 %). The respondents were relatively well-experienced as the mean number of years at their current workplace was 8 years, and the mean “years of experience in community elderly care” was over 15 years (see Table 4).

### The competence of nursing staff in community elderly care

The competence measurement revealed that nursing staff in Norwegian community elderly care possess competence in all competence variables measured, however, to a varying degree. Table 5 reports the results from the analysis of variance of nursing staff’s competence levels.

**Table 5:** Mean level of competence in nursing staff groups (ANOVA)

	RNs	ANs	Assistants	Max^a^	*p*		RNs	ANs	Assistants	Max^a^	*p*
Palliative care	26.57	23.14	15.92	32	.000	Body mechanics	14.68	15.21	13.86	20	.000
Acute help	3.47	2.35	1.98	5	.000	Simple procedures	4.27	4.41	3.54	8	.000
Wound care	27.09	27.11	22.64	32	.000	Make oneself understood	7.48	7.37	7.50	8	.442
Nursing measures	6.61	7.31	6.62	16	.002	Electronic communication	2.66	1.99	1.96	8	.000
Def. in sight and hearing	13.05	13.44	12.19	16	.000	Patient assessment	13.46	13.17	9.09	16	.000
Fall prevention	1.16	1.21	1.25	3	.871	Skills sum variable	101.01	89.72	72.82	176	.000
Diabetes treatment	2.75	2.31	1.24	3	.000	Coop./physician & next-of-kin	20.95	18.69	17.35	26	.000
Medication calculation	1.90	0.36	0.28	2	.000	Not reach co-workers	8.79	9.31	8.45	24	.238
Involving physician	3.91	2.69	2.31	5	.000	Coop./ANs & assistants	15.29	16.42	16.90	18	.000
Health promotion	10.09	9.91	9.01	12	.000	Coop./documentation	9.44	10.20	11.14	12	.000
New palliative measures	6.84	4.27	2.74	12	.000	Coop./leader	10.80	10.69	10.98	13	.628
Knowledge sum variable	103.39	93.90	75.88	138	.000	Coop./RNs	8.76	8.77	8.53	9	.039
Patient observations	10.79	9.74	6.78	16	.000	Communication	11.39	11.39	10.95	12	.015
Advanced procedures	21.03	13.87	12.91	60	.000	Attitudes towards elderly	10.64	10.58	10.05	12	.006
Nursing documentation	8.38	6.24	4.08	16	.000	Personal attributes sum variable	95.96	96.09	93.17	126	.056
Treatment	18.20	17.44	11.37	24	.000	Total competence sum variable	283.33	247.68	198.83	440	.000

^a^Maximum score. Knowledge sum variable: F = 114.32, df = 2, *p* = .000, Skills sum variable: F = 85.94, df = 2, *p* = .000, Personal attributes sum variable: F = 2.9, df = 2, *p* = .056, Total competence sum variable: F = 58.61, df = 3, *p* = .000

The main trend was that RNs displayed more competence than ANs, who again displayed more competence than assistants, but there were exceptions, e.g. ANs scored higher than RNs on “nursing measures” and “simple procedures”. Another trend was that the mean scores were substantially lower than the maximum score for quite a few sum variables, e.g. “nursing measures”, “advanced procedures”, and “nursing documentation”.

### Predictors of competence as measured

The linear model chosen explained 30 % of the variance in the “total competence sum-variable” (Table 6).

**Table 6:** Linear model of predictors of total competence

	b (CI)	SE B	β	*p*
Constant	303.81 (284.91, 322.71)	9.63		*P* = .00
Professional group: ANs^a^	−29.48 (−36.56,−22.39)	3.61	−.27	*P* = .00
Professional group: Assistants^a^	−77.44 (−91.16,−63.73)	6.99	−.38	*P* = .00
Professional group: Others^a^	−87.57 (−107.71,−67.42)	10.26	−.25	*P* = .00
Workplace: Nursing home^b^	10.72 (3.97, 17.46)	3.44	.10	*P* = .00
Workplace: other^b^	−15.82 (−26.73,−4.91)	5.56	−.09	*P* = .01
Type of position	−2.04 (−16.64, 12.56)	7.44	−.01	*P* = .78
Size of position	14.13 (−0.77, 29.03)	7.59	.06	*P* = .06
Years at current workplace	0.24 (−0.26, 0.74)	0.25	.03	*P* = .34
Years in community care	0.24 (−0.20, 0.68)	0.22	.05	*P* = .28
Age	−0.99 (−1.33,−0.64)	0.18	−.21	*P* = .00
Gender	−0.89 (−16.26, 14.48)	7.83	.00	*P* = .91

Confidence intervals (CI) and standard errors (SE) based on 1000 bootstrap samples

R^2^ = .30, ΔR^2^ = .30 (*p* = .00), F = 34.30, *p* = .000, ^a^Reference group is RNs, ^b^Reference group is Home care services

The F-ratio =34.30 with a *p* < .00, which means that our regression model predicted competence-level significantly better than no model of competence-level. The linear model showed that ANs scored 29.48 points less than an RN (*p* = .00), assistants scored 77.44 points less than an RN (*p* = .00), other professionals scored 87.57 points less than an RN (*p* = .00), respondents working in nursing homes scored 10.72 points more than respondents working in home care services (*p* = .00), respondents working in other services for elderly scored 15.82 points less than respondents working in home care services (*p* = .01), and that age influence competence negatively (*p* = .00). The type of position, size of position, years at current work place, years in community elderly care, and gender did not have significant effect on the model.

## Discussion

### Representability

The distribution of nursing staff groups in our sample (RNs: 35 %, ANs: 52 %, assistants: 9 %, and others: 4 %) match the actual number of RNs (34 %) and ANs (58 %) in the population well [29]. In an attempt to grasp the diversity of Norwegian community elderly care, the municipalities we chose for the trial represent different geographical and demographical parts of Norway, are run by different political parties, and invest differently in competence development. The results for RNs and ANs can therefore be assumed to have transferability to similar nursing staff in other Norwegian municipalities. The number of assistants that responded to the NOP-CET is, however, much lower than the number estimated to be up to 28 % in some municipalities [28], and we can therefore expect the results from the assistants to be biased. The assistants that responded could be particularly competent and therefore willing to fill out the questionnaire, or they could be more computer-able than other assistants. Although some years back, MacDonald et al. [46] found that 30 % of assistants had no computer experience, implying that the electronic response format could have been a barrier to the response of assistants. Although the respondents were informed that they could probably not respond to all items, another reason for the low participation of assistants could be that assistants found it intimidating or discouraging to complete a questionnaire in which they expected to fall short in terms of results. Future use of the NOP-CET should therefore include the alternative of filling out a paper version, and find other ways in which assistants might be encouraged to participate.

### The competence profile of the nursing staff

All nursing staff had some competence in the competence variables measured. As expected, the RNs scored higher than ANs and assistants on the majority of the competence variables. This is reassuring, as RNs have the highest education and are responsible for the nursing care in Norwegian community elderly care. There were, however, variables on which ANs and/or assistants scored higher than RNs: nursing measures (ANs/assistants get a score for answering “consult an RN”, whereas RNs do not), deficiencies in sight and hearing, body mechanics, simple procedures, make oneself understood, personal attributes concerning reaching co-workers, cooperation with ANs and assistants, cooperation concerning documentation, cooperation with leader and RNs, and the personal attributes sum-variable.

Indeed, it could be that ANs who are generally well experienced in community elderly care have higher competence than RNs (mean years of experience for ANs in community elderly care was 15). ANs work mostly with patient-direct work and may therefore be more competent in such areas. Another explanation is that RNs may be more self-critical to their own competence than ANs and assistants (on self-evaluation items). In a review of the effectiveness of self-assessment Colthart et al. [47] found that ability and experience appears to affect self-assessment, meaning that competent practitioners are more accurate in their self-assessment than individuals who lack competence.

What is equally interesting about the results from the competence measurement is that on no competence variables do any of the nursing staff groups reach the maximum score. On some variables there is even a large gap between the maximum score and the achieved mean score, e.g. on nursing measures, new palliative measures, patient observations, advanced procedures, nursing documentation, electronic communication, and not being able to reach co-workers. As the NOP-CET measures competence necessary to provide safe care to frail, older patients this is worrying. Older patients in community elderly care are as described characterised by multi-morbidity, polypharmacy, and/or cognitive failure, which requires that adequate nursing care and treatment is initiated without delay [19]. The results from this survey indicate that nursing staff as a group does not have sufficient competence to secure the required care and treatment of older patients as they lack basic nursing competence in observation, systematic assessment, initiating nursing measures, performing advanced procedures, documenting their work, and cooperating with co-workers when required (cannot reach them). This survey indicates that there are several areas of competence that need to be improved in order to achieve safe patient care in community elderly care. A recent report found that the Norwegian municipalities had not offered or facilitated sufficient competence development to their nursing staff in accordance with the increasing complexity in current community elderly care [30]. Therefore a large competence challenge is facing the municipalities as they are required by law to provide safe care to people in need of health care in accordance with their needs [48].

Still, the indications of inadequacy in competence must be considered with precautions as a cut-off for minimum acceptable score has not yet been set. Future research into competence measurement of nursing staff should therefore establish the lowest clinically acceptable score for the nursing staff group as a whole, and for each of the three nursing staff groups separately. This exercise could help municipalities to differentiate better between groups, to understand which group is competent for which task and which group of patients, and to evaluate this continually. Research has shown that there is a link between adverse events in nursing care and competence level [26, 49], and one way to evaluate quality of care is to secure that those who provide care and treatment are sufficiently competent to do so.

It is uplifting that competence as measured results in the expected pattern of RNs having more competence than ANs who again have more competence than assistants. This is yet another sign of validity, i.e. known-group validity [45]. In our conceptualisation of competence we also pointed at the importance of collective competence; that nursing staff as a whole needs to be competent to provide safe health care to older patients. In this light the varying competence levels as depicted in Table 5 can be considered complimentary to one another, and may assure that the sum (i.e. collective competence) is more than its parts.

### Influences on competence

The results from the regression model showed that professional group affiliation, working place, and age influenced competence level. These variables explain 30 % of the variance in competence. One could, however, expect that education and training would have more impact on competence than what is shown. This result could be influenced by the element of self-assessment that the NOP-CET incorporates.

Gordon [50] defines valid self-assessment as judging one’s performance against appropriate criteria, and accurate self-assessment as gaining reasonable concurrence between self-acclaimed and other, validated measures of competence. In this definition the importance of appropriate criteria against which to judge one’s own competence is central. Thus, an explanation for the relatively low influence education and training has on competence could be that nursing staff have poor criteria against which they can judge their own competence. Nursing staff in Norwegian nursing homes and home care services handle very similar tasks, and are expected to care for most patients, regardless of group affiliation. Haukelien [31] found that the relatively low competence available in community elderly care is reinforced by an attitude of “pulling the load together”, which entails that everybody must do all tasks in order to keep it going and that RNs with the highest competence therefore do not put all their competence to use. As community elderly care is increasingly taking on more patients in a complex, frail state, we believe that role differentiation should be much clearer, and role descriptions of expected competence should be created.

The regression model showed that staff in nursing homes scored 10 points more than staff in home care services, which we can interpret as staff in nursing homes have more competence than staff in home care services. In light of our conceptualisation of competence, this result is understandable because staff in home care services work mostly on their own in patients’ homes and can rely less on collective, relational competence. It is also worrying, as patients in home care are more reliant on the competence of individuals than in nursing homes where there is more staff present at all times. An implication of this could therefore be to take a closer look at what competence home care staff is lacking and how to alleviate the competence gap between nursing homes and home care services.

Finally, age is shown to have a negative impact on competence, meaning that the older respondents scored lower than younger respondents. This finding is likely due to the generally higher age of ANs than RNs, but could also be caused by a cohort effect in terms of education and training, meaning that the oldest respondents have not been able to stay up-to-date due to the general lack of competence development. This finding could have the implication of giving staff who have worked for many years the opportunity to upgrade their competence and keep up-to-date on developments in the field of community elderly care.

## Conclusion

When assessing all staff in relation to the expected competence level, we are in a position to say how competence differs between nursing staff groups; and give leaders in community elderly care a tool to work systematically with developing the competence needed to provide safe care to older patients. In this survey we found that overall RNs are more competent than ANs and assistants, but that the two latter groups also score higher than RNs in some areas. However, as collective competence is an important prerequisite for how we have measured competence, the varying competence levels may work complimentary and thereby assure sufficient competence levels. A more detailed exploration of whether the shown competence levels of nursing staff are actually sufficient is, however, needed. Future research should therefore establish the lowest clinically acceptable competence levels for community-based nursing staff.

We found that a multiple linear model predicts 1/3 of the variation in competence, thus many factors which could explain differences in competence-levels other than education/training, workplace and age are left to be explored. A reason for the relatively low influence of education and training on competence could be the diffuse roles that nursing staff have in community elderly care, implying that they have poor standards against which to judge their own competence. Clearer role descriptions for all groups of nursing staff are recommended.

## Abbreviations

NOP-CET: Nursing older people – competence evaluation tool
RN: Registered nurse
AN: Assistant nurse

## References

[1] Boerma GW, Saltman RB, Rico A, Boerma GW (2006). Coordination and integration in European primary care. Primary Care in the driver’s Seat? Organizational Reform in European Primary Care.

[2] Selbæk G (2008). Behavioural and Psychological Symptoms of Dementia in Norwegian Nursing Homes - Prevalence, Course and Association With Psychotropic Drug use.

[3] Pedersen PB, Kolstad A (2009). De-institutionalisation and trans-institutionalisation - changing trends of inpatient care in Norwegian mental health institutions 1950-2007. Int J Ment Heal Syst.

[4] Gautun H, Hermansen A (2011). Geriatric care under pressure. Municipal health and care services for the elderly [In Norwegian].

[5] Genet N, Boerma W, Kroneman M, Hutchinson A, Saltman RB (2012). Home Care Across Europe: Current Structure and Future Challenges.

[6] Valderas JM, Starfield B, Sibbald B, Salisbury C, Roland M (2009). Defining comorbidity: implications for understanding health and health services. Ann Fam Med.

[7] Bayliss EA, Steiner JF, Fernald DH, Crane LA, Main DS (2003). Descriptions of barriers to self-care by persons with comorbid chronic diseases. Ann Fam Med.

[8] Wolff JL, Starfield B, Anderson G (2002). Prevalence, expenditures, and complications of multiple chronic conditions in the elderly. Arch Intern Med.

[9] Hajjar ER, Cafiero AC, Hanlon JT (2007). Polypharmacy in elderly patients. Am J Geriatr Pharmacother.

[10] Jyrkka J, Vartiainen L, Hartikainen S, Sulkava R, Enlund H (2006). Increasing use of medicines in elderly persons: a five-year follow-up of the Kuopio 75 + Study. Eur J Clin Pharmacol.

[11] Hovstadius B, Hovstadius K, Astrand B, Petersson G (2010). Increasing polypharmacy - an individual-based study of the Swedish population 2005-2008. BMC Clin Pharml.

[12] Nyborg G, Straand J, Brekke M (2012). Inappropriate prescribing for the elderly--a modern epidemic?. Eur J Clin Pharmacol.

[13] Steinman MA, Handler SM, Gurwitz JH, Schiff GD, Covinsky KE (2011). Beyond the prescription: medication monitoring and adverse drug events in older adults. J Am Geriatr Soc.

[14] Mannesse CK, Derkx FH, de Ridder MA, Man in ’t Veld AJ, van der Cammen TJ (2000). Contribution of adverse drug reactions to hospital admission of older patients. Age Ageing.

[15] Ebbesen J, Buajordet I, Erikssen J, Brors O, Hilberg T, Svaar H (2001). Drug-related deaths in a department of internal medicine. Arch Intern Med.

[16] Roen I, Selbaek G, Kirkevold O, Bergh S, Engedal K (2013). The Prevalence of Dementia and Neuropsychiatric Symptoms in Patients at Admission to Norwegian Nursing Homes. 16th International Congress of the International Psychogeriatric Association.

[17] Selbaek G, Engedal K, Bergh S (2013). The prevalence and course of neuropsychiatric symptoms in nursing home patients with dementia: a systematic review. J Am Med Dir Assoc.

[18] Selbaek G, Engedal K, Benth JS, Bergh S (2014). The course of neuropsychiatric symptoms in nursing-home patients with dementia over a 53-month follow-up period. Int Psychogeriatr.

[19] Rytter L, Jakobsen HN, Ronholt F, Hammer AV, Andreasen AH, Nissen A (2010). Comprehensive discharge follow-up in patients’ homes by GPs and district nurses of elderly patients. A randomized controlled trial. Scand J Prim Health Care.

[20] Norwegian Ministry of Education and Research (2012). Meld. St. 13 (2011-2012) Utdanning for velferd. Samspill i praksis. Norwegian Education for welfare. Coordination in practice.

[21] Randolph P, Hinton J, Hagler D, Mays MZ, Kastenbaum B, Brooks R (2012). Measuring competence: collaboration for safety. J Cont Educ Nurs.

[22] Jarman B, Gault S, Alves B, Hider A, Dolan S, Cook A (1999). Explaining differences in English hospital death rates using routinely collected data. Br Med J.

[23] Pronovost P, Angus D, Dorman T, Robinson K, Dremsizov T, Young T (2002). Physician staffing patterns and clinical outcomes in critically ill patients: a systematic review. J Am Med Assoc.

[24] Desjardins F, Cardinal L, Belzile E, McCusker J (2008). Reorganizing nursing work on surgical units: a time-and-motion study. Nurs Leadersh.

[25] Hendrich A, Chow MP, Skierczynski BA, Lu Z (2008). A 36-hospital time and motion study: how do medical-surgical nurses spend their time?. Perm.

[26] Bostick JE, Rantz MJ, Flesner MK, Riggs CJ (2006). Systematic review of studies of staffing and quality in nursing homes. J Am Med Dir Assoc.

[27] Spilsbury K, Hewitt C, Stirk L, Bowman C (2011). The relationship between nurse staffing and quality of care in nursing homes: a systematic review. Int J Nurs Stud.

[28] Romøren TI, Torjesen DO, Landmark B (2011). Promoting coordination in Norwegian health care. Int J Integr Care.

[29] Statistics Norway. Tabell: 09934: Årsverk innanfor pleie- og omsorgstenestene, etter utdanning. Pleie- og omsorgstenester. [In Norwegian] [Table: 09934: Full-time equivalent employees in municipal care services by education]. www.ssb.no: Statistics Norway 2014 01.01.2014.

[30] Gautun H, Syse A. The Coordination Reform. How do community health services receive the increased amount of patients submitted from hospitals? [In Norwegian]: NOVA – Norwegian Social Research 2013.

[31] Haukelien H (2013). Omsorg og styring: kjønn, arbeid, og makt i velferdskommunen. [In Norwegian] [Nursing and management: gender, labour, and power in the welfare municipality].

[32] Baldwin J, Roberts JD, Fitzpatrick JI, While A, Cowan DT (2003). The role of the support worker in nursing homes: a consideration of key issues. J Nurs Manag.

[33] Furåker C (2012). Registered Nurses’ views on competencies in home care. Home Health Care Manag Pract.

[34] Sørbye LW, Grue EV, Vetvik E (2009). Kunnskap om svikt i tjenester til skrøpelige eldre. Nyere forskning relatert til helse- og sosialtjenesten. [In Norwegian] [Knowledge concerning adverse events in services to frail elderly. New research related to municipal health care].

[35] Norwegian Board of Health Supervision (2009). Rapport fra tilsyn med hjemmetjenester til pasienter i hjemmesykepleien i Oslo kommune bydel Gamle Oslo. Norwegian Report from Supervision of Home Care Services to Patients in Oslo Municipality District Gamle Oslo.

[36] Huseby BM, Paulsen B (2009). Eldreomsorgen i Norge: Helt utilstrekkelig - eller best i verden?. [In Norwegian] [Care for elderly in Norway: Completely inadequate - or the best in the world?].

[37] Norwegian Board of Health Supervision (2014). Oppsummering av satsinga på tilsyn med helse- og omsorgstenester til eldre 2009–2012. [In Norwegian] [Summary of supervision of health and care services for elderly people 2009-2012].

[38] Norwegian Board of Health Supervision (2013). Oppsummering av landsomfattende tilsyn i 2011 og 2012 med tvungen helsehjelp til pasienter i sykehjem. Tvil om tvang. [In Norwegian] [Doubt about coercion. Summary of countrywide supervision of compulsory health care for patients in nursing homes in 2011 and 2012].

[39] Garside JR, Nhemachena JZ (2012). A concept analysis of competence and its transition in nursing. Nurse Educ Today.

[40] Eraut M (1994). Developing Professional Knowledge and Competence.

[41] Edwards A (2010). Being an Expert Professional Practitioner.

[42] Bing-Jonsson PC, Bjørk IT, Hofoss D, Kirkevold M, Foss C (2013). Instruments measuring nursing staff competence in community health care. A systematic literature review. Home Health Care Manag Pract.

[43] Bing-Jonsson PC, Bjørk IT, Hofoss D, Kirkevold M, Foss C (2015). Competence in advanced older people nursing: development of ‛Nursing older people - Competence evaluation tool’. Int J Older People Nursing.

[44] Bing-Jonsson PC, Hofoss D, Kirkevold M, Bjørk IT, Foss C (2015). “Nursing older people - competence evaluation tool”: development and psychometric evaluation. J Nurs Meas.

[45] Field A (2013). Discovering Statistics Using IBM SPSS Statistics.

[46] MacDonald CJ, Stodel EJ, Casimiro L (2006). Online dementia care training for healthcare teams in continuing and long-term care homes: a viable solution for improving quality of care and quality of life for residents. Int J E-Learn.

[47] Colthart I, Bagnall G, Evans A, Allbutt H, Haig A, Illing J (2008). The effectiveness of self-assessment on the identification of learner needs, learner activity, and impact on clinical practice: BEME Guide no. 10.. Medical Teach.

[48] Norwegian Ministry of Health and Care Services (2011). Prop. 91 L (2010-2011) Lov om kommunale helse- og omsorgstjenester m.m. (helse- og omsorgstjenesteloven). Norwegian Proposal 91 L to the Storting: Act on Health and Long-Term Care.

[49] Spilsbury K, Meyer J (2001). Defining the nursing contribution to patient outcome: lessons from a review of the literature examining nursing outcomes, skill mix and changing roles. J Clin Nurs.

[50] Gordon MJ (1991). A review of the validity and accuracy of self-assessments in health professions training. Acad Med.

